# Inducible microRNA-200c decreases motility of breast cancer cells and reduces filamin A

**DOI:** 10.1371/journal.pone.0224314

**Published:** 2019-11-20

**Authors:** Bojan Ljepoja, Christoph Schreiber, Florian A. Gegenfurtner, Jonathan García-Roman, Bianca Köhler, Stefan Zahler, Joachim O. Rädler, Ernst Wagner, Andreas Roidl

**Affiliations:** 1 Pharmaceutical Biotechnology, Department of Pharmacy, Ludwig-Maximilians-Universität München, Munich, Germany; 2 Faculty of Physics and Center for NanoScience, Ludwig-Maximilians-Universität München, Munich, Germany; 3 Pharmaceutical Biology, Department of Pharmacy, Ludwig-Maximilians-Universität München, Munich, Germany; University of Nebraska Medical Center, UNITED STATES

## Abstract

Cancer progression and metastases are frequently related to changes of cell motility. Amongst others, the microRNA-200c (miR-200c) was shown to maintain the epithelial state of cells and to hamper migration. Here, we describe two miR-200c inducible breast cancer cell lines, derived from miR-200c knock-out MCF7 cells as well as from the miR-200c-negative MDA-MB-231 cells and report on the emerging phenotypic effects after miR-200s induction. The induction of miR-200c expression seems to effect a rapid reduction of cell motility, as determined by 1D microlane migration assays. Sustained expression of miR200c leads to a changed morphology and reveals a novel mechanism by which miR-200c interferes with cytoskeletal components. We find that filamin A expression is attenuated by miRNA-200c induced downregulation of the transcription factors c-Jun and MRTF/SRF. This potentially novel pathway that is independent of the prominent ZEB axis could lead to a broader understanding of the role that miR200c plays in cancer metastasis.

## Introduction

Metastasis, i.e. the nesting of tumor cells in adjacent tissues and even distant organs, is one of the most malicious aspects of cancer, causing nine out of ten cancer deaths [1]. While primary tumors often can be treated well, the uncontrollable spread of cancer cells remains a major challenge in most clinical settings. One prevalent example for risks of metastatic cancers are tumors of the breast, which show a clear association between metastasis and survival of patients [2, 3]. While the primary breast carcinomas show rather good resectability due to their location, the cancer often has reached distant organs before the primary tumor was detected. Progress in understanding the disease has been made by identifying certain subtypes of breast tumor cells which inherit particularly high metastatic potentials [4]. However, current studies show a rise in incidence of metastatic breast cancer [5]. Therefore, still more and deeper insights into the key regulators of migratory and metastatic processes are needed.

Epithelial to mesenchymal transition (EMT) is often regarded as one of the most important steps in the initiation of migration and thus the onset of invasion and metastasis of tumors [6–8]. While EMT can be influenced by multiple cellular processes, RNA interference by microRNAs (miRNAs) was shown to be a direct and important regulatory mechanism [9].

In general, miRNAs are small, non-coding RNAs, influencing the translation of multiple fundamental cellular processes like metabolism, proliferation and cellular organization. Even small changes in miRNA expression patterns can have tremendous impact on the cell fate and can prompt towards various malignancies or even be the root cause of those [10–12]. One miRNA family with important implications in cancer is the miR-200 family, consisting of miR-200a, miR-200b, miR-141, miR-429 and miR-200c. While all members have demonstrated effects in the regulation of cancer processes, miR-200c is the family member which unifies well investigated associations in the most important cancer pathways, like the inhibition of chemoresistance[13–15], regulation of metabolic activity[16–18] and also in epithelial-to-mesenchymal transition (EMT) and thus potentially cancer cell metastasis [13, 18–22].

MiR-200c’s role in the regulation of EMT is based on its stabilizing effect on the expression of E-cadherin by preventing the inhibition of E-cadherin by ZEB1 and ZEB2 (Zinc finger E-box-binding homeobox members 1 and 2). Previous studies have shown that the introduction or re-expression of miR-200c *in vitro* reverses the mesenchymal phenotype of cancer cells, i.e. leading to EMT reversion, termed MET (mesenchymal to epithelial transition) [19, 22].

Although EMT may be one of the main pathways of metastasis induced by the loss of miR-200c, the metastatic capabilities of tumor cells also rely on multiple other mechanisms. Interestingly, miR-200c was shown to influence other migratory pathways, for example by regulation of fibronectin secretion and moesin expression or by targeting the SRF-regulating proteins FHOD1 and PPM1F [20, 23, 24] as well as Sec23a [25].

To further investigate the function of miR-200c, we previously generated a genomic knock-out (KO) of miR-200c in MCF7 breast cancer cells [26]. The resulting KO phenotype showed increased migration, even of the epithelial and usually low-migrating MCF7 cells. A pooled proteomic analysis revealed several common differentially regulated proteins, half of which are attributed to the regulation of migratory processes. From this set of proteins, promising candidates were chosen for further investigation. One protein of particular interest was Filamin A, a member of the filamin protein family, which are known building blocks of the cytoskeleton and thereby involved in many intracellular and migratory processes [27]. Filamins, and especially filamin A, function as important actin filament crosslinkers, facilitating actin-actin interactions, but also actin-connections to membrane bound proteins and intracellular signaling macromolecules [27, 28]. Previous studies described the role of filamin A in the regulation of cell migration [29]. However, a systematic investigation of miR-200c mediated expression of filamin A and concomitant changes in migration has not yet been conducted.

In this study, we generated two different inducible miR-200c breast cancer cell line models, derived either from mesenchymal MDA-MB-231 cells or the miR-200c knock-out of the epithelial MCF7 cells, respectively. By doxycycline induction, we investigated the effect of increased miR-200c expression on morphological changes and motility. We used a micro-pattern based 1D migration assay, as described previously by Schreiber *et al*. [30] to get a multiparameter quantification of cell motility. We also found strong indications of a regulatory network of miR-200c and FLNA in both breast carcinoma models. This pathway, which is independent of the ZEB-expression of the cells, may point towards an important further function of miR-200c in impeding cancer metastasis.

## Results

### The migratory potential of MDA-MB-231 cells decreases after miR-200c induction

To investigate the effect of miRNA-200c induction on the metastatic potential of cells, we performed an in-depth analysis of single cell migration.

**Fig 1:**
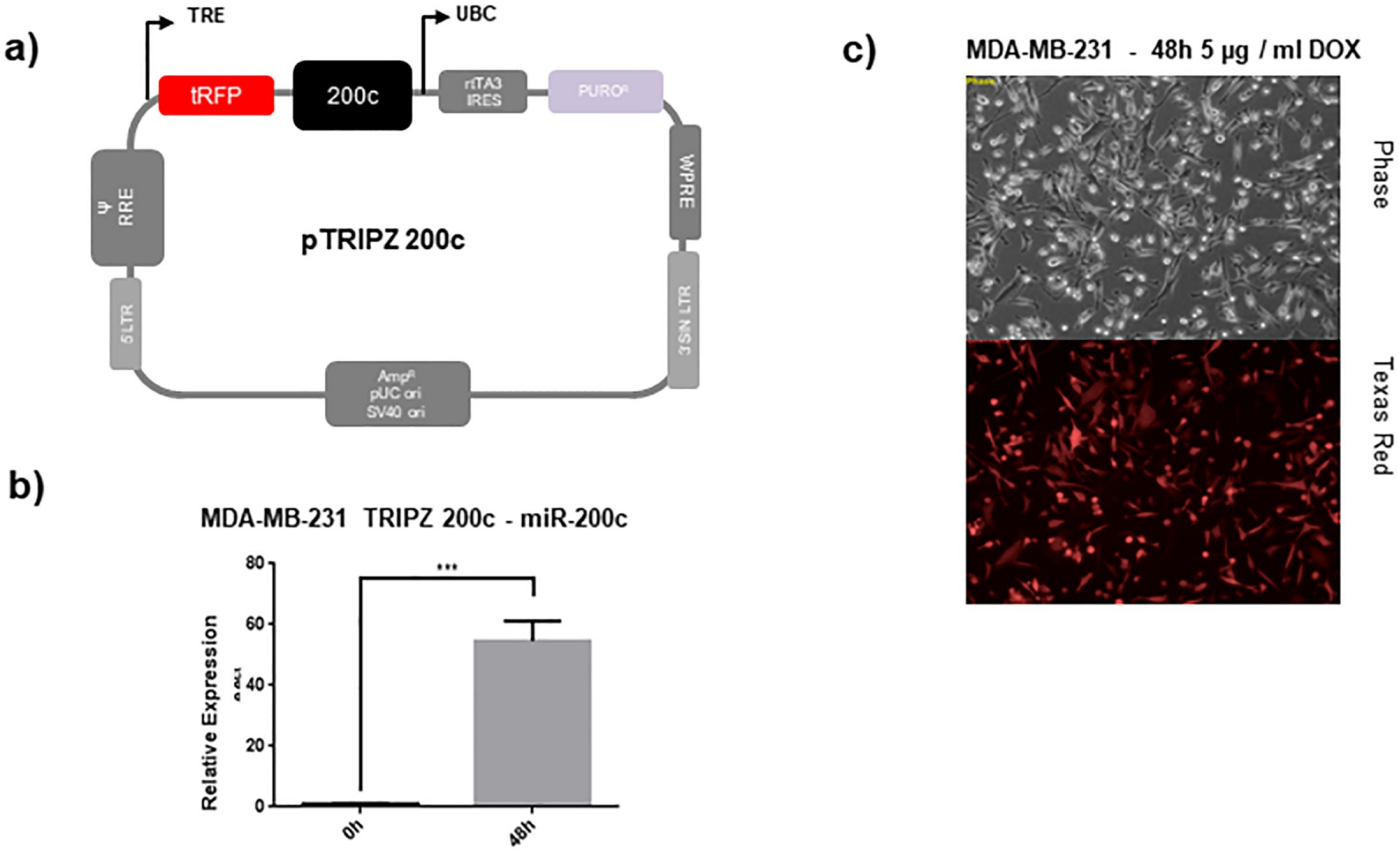
Inducible miR-200c construct with RFP reporter. (a) Description of the inducible pTRIPZ-200c construct (b) and verification of functional transduction in MDA-MB-231 cells by induction of the RFP reporter tag by 5 μg / ml doxycycline for 48 h. (c) Expression analysis by RT-qPCR of miRNA-200c after induction with 5μg / ml doxycycline after 48 h.

Therefore, the miR-200c non-expressing, highly migratory MDA-MB-231 cell line [17] was chosen for stable transduction with a TET-off construct containing either miR-200c or a scrambled control, resulting in the MDA-MB-231 TRIPZ-200c or MDA-MB-231 TRIPZ-Ctrl cells. Treatment with doxycycline for 48 h showed a reliable and controllable induction of miR-200c expression as well as of the RFP reporter tag (Fig 1a and 1b).

With these cell line models, we performed a multi-parameter analysis of motility, utilizing our 1D migration system of ring-shaped micro-lanes. Compared to other common migratory assays, the real time tracking of the 1D migration allowed for analysis of a higher number of single cells and for generation of a multi-parameter migratory fingerprint, i.e. cell velocity, cell persistence, cell resting times, cell run times and the run fraction, at single cell and population level.

**Fig 2:**
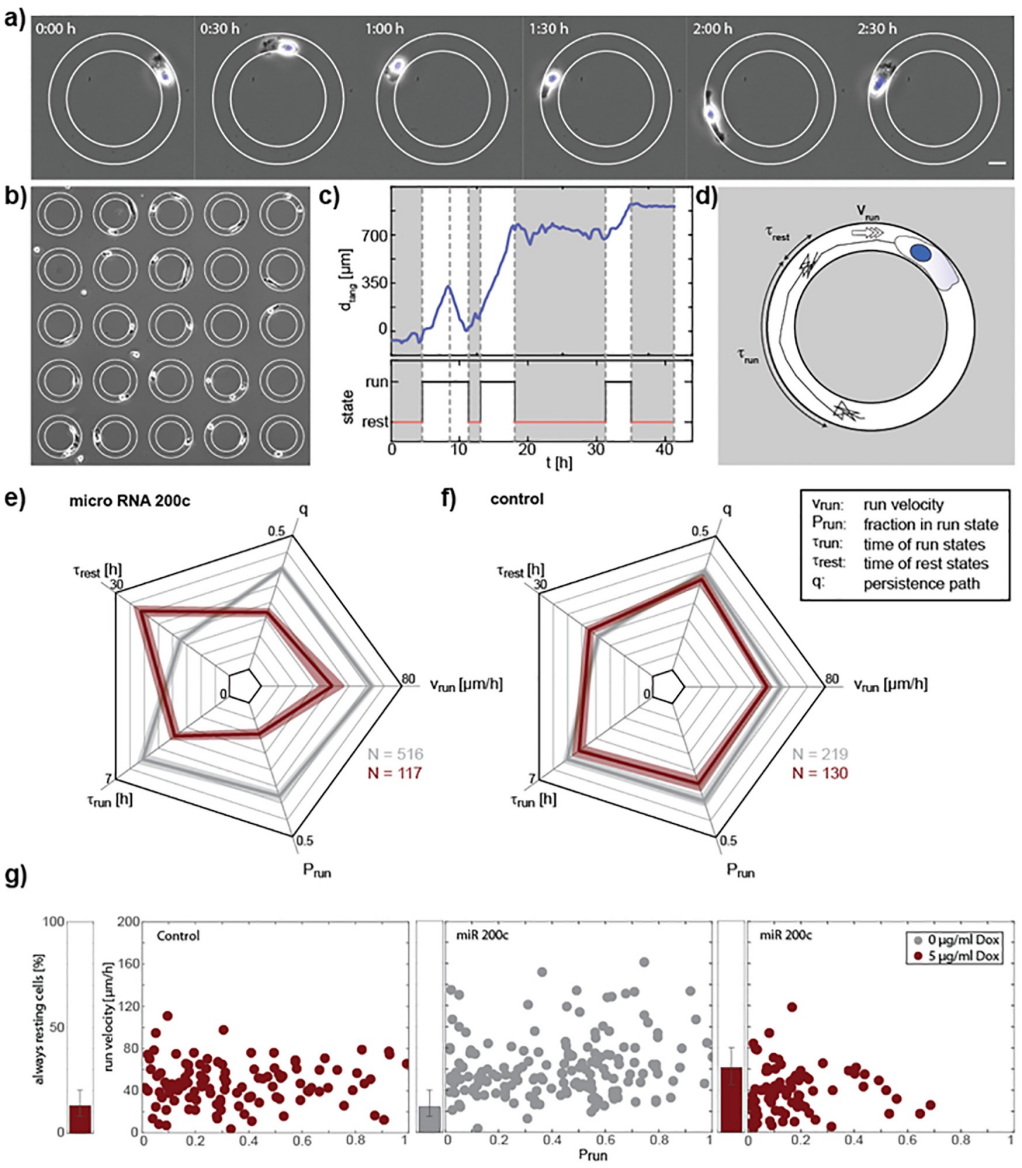
miR-200c induction decreases migration of MDA-MB-231 cells as shown in the 1D migration assay. (a) Phase contrast images of a MDA-MB 231 cell migrating on a ring shaped micro-lane. The ring is coated with fibronectin (edges marked in white) the surrounding is passivated with PEG. The scale bar is 20 μm. (b) Array of ring shaped micro lanes. Only rings that are occupied by one single cell are evaluated. (ring diameter 150 μm) (c) Angular position of one exemplary cell over time with classification into run and rest states. (d) Drawing of a cell track. Cell motion can be separated in run states with ballistic motion and rest states with random motion. The characteristic duration of run and rest states *τ*_run_, *τ*_rest_ as well as the velocity in the run state *v*_run_ are evaluated. (e, f) Multi-parameter analysis of cell motility of cell populations. Motility of cells is measured 48h after induction with 5 μg / ml doxycycline (red). For MDA-MB-231 TRPZ-200c cells (f) a clear reduction of cell motility can be seen in all of the 5 parameters compared to no induction (grey). For MDA-MB-231 TRPZ-Ctrl cells (h) no big effects on motility are observed with adding doxycycline. N is the number of cells analyzed. (g) Single cell analysis of *P*_run_ and *v*_rest_ for the data shown in e, f) where each dot represents a single cell. One cell population is spread over a large range of velocities and fraction of time in the run state. Induction of miR 200c is causing a shift to slower velocities and less time spent in the rest state. (error bars in g and h indicate standard errors exept for *τ*_run_, *τ*_rest_ where it’s CI of 99% of the fit).

For this purpose, cells were seeded on arrays of fibronectin-coated ring micropatterns and were observed using time-lapse microscopy (Fig 2a and 2b). We found that the 1D cell motion was divided into distinct run states, where cells moved persistently in one direction, and rest states with no or random wiggling motion [30] (Fig 2c). This two state analysis resulted in characteristic parameters quantifying cell motility (Fig 2d). By discriminating between run and rest states we made sure that the velocity was only evaluated when cells were actually migrating (*v*_run_). Furthermore, we analyzed the typical lifetime of run and rest states *τ*_run_ and *τ*_rest_, which were exponentially distributed. This allowed distinguishing between the stability of the run state, given by *τ*_run_ and the ability of cells to establish polarization indicated by *τ*_rest_. For a comprehensive overview of the different motility parameters, spider-plots were generated (Fig 2e and 2f). As expected, the doxycycline induction in the MDA MB-231 TRIPZ-Ctrl cells showed no significant effects compared to the uninduced cells, while miR-200c induction distinctly changed the migratory behavior of the cells. The run velocity and the typical duration of a run state were significantly decreased, whereas the typical duration of a rest state was increasing. The strongest effect was observed in the fraction of time that cells spent in the run state, *P*_run_, which decreased by a factor of three. Thus, induction of miR-200c expression affected the polarization of cells leading to longer rest states and a decreased persistence of the run states. To show that the decrease of persistence of the cell motion was also visible without the segmentation into run and rest states, we evaluated the persistence path q, which is given by the effective maximum displacement of a cell divided by the actual length of the trajectory, as described in Maiuri *et al*. [31]. The described effects were visualized in a sample of a Ctrl vs a miR-200c induced cell, as shown in the supplemental movie (S1 Movie). On single cell level, a broad distribution of run velocities and a huge variance in the fraction of time spent in the run state was observed (Fig 2g). With increasing miR-200c expression, the distribution narrowed, and the average velocity was decreasing as well as the time cells spent in run states. Furthermore, the fraction of cells that remained in the rest states for the time of the whole experiment increased by almost a factor of three.

Taken together, our data shows that the induced miR-200c expression resulted in a reduced motility in all five migratory parameters and, hence, an overall decreased migratory potential. The observed process must be independent of the well investigated miR-200c and ZEB1/2 induced EMT mechanisms [19]; due to the fact that MDA-MB-231 cells are not expressing E-cadherin [32–34].Our findings therefore suggest a novel mode of miR-200c acting on migration.

### miR-200c induction changes the 3D morphology

**Fig 3:**
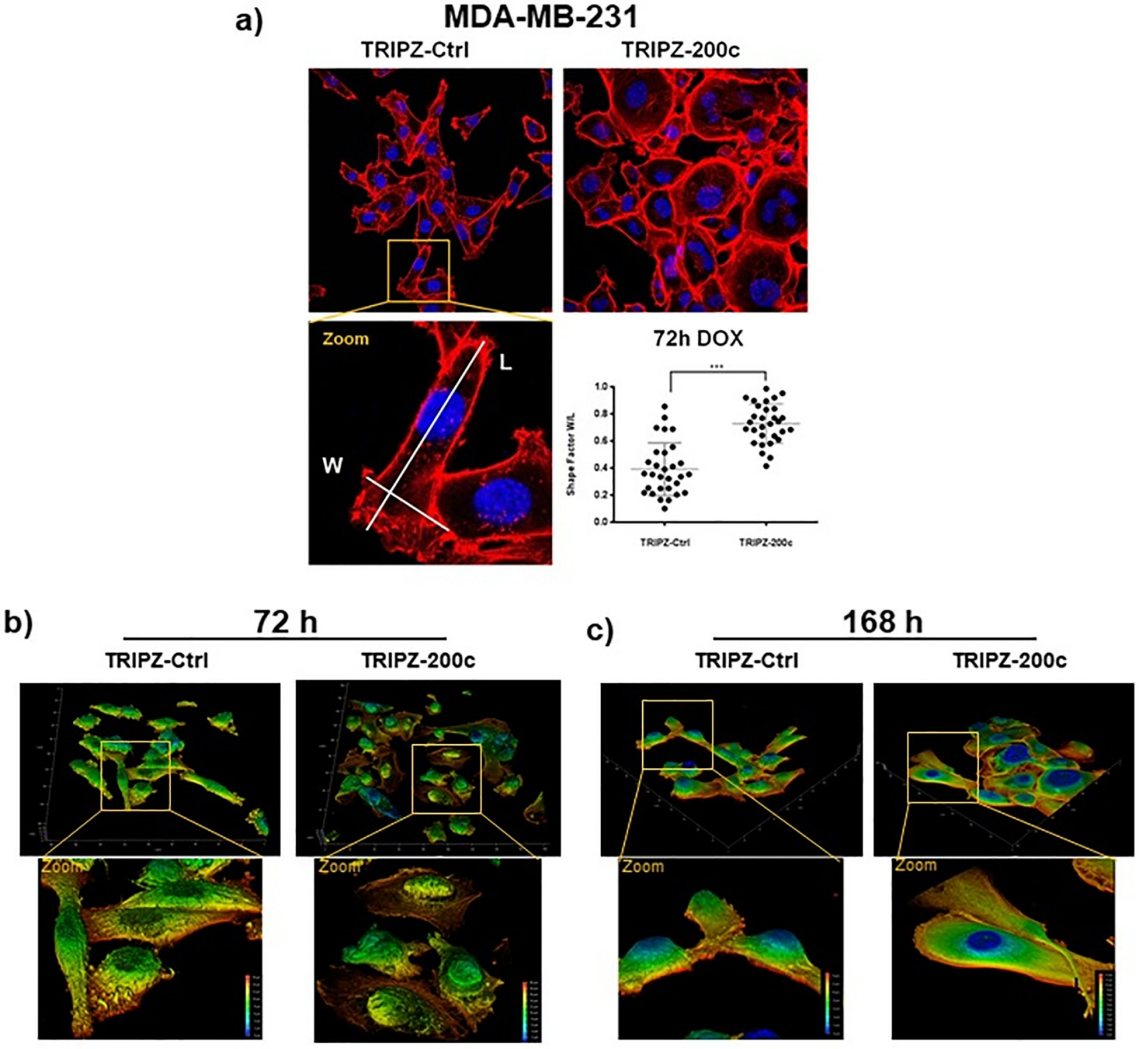
Overexpression of miR-200c induced fast morphological changes in MDA-MB-231 cells. (a) Fluorescence staining of the actin cytoskeleton by phalloidin (red) and nuclei (blue) in MDA-MB-231 decreased spindle-like phenotype after induction miR-200c, as shown by significant changes in the shape factors (N = 30; error bars are SD; *** p > 0.001). (b, c) Renderings of z-stacked immunofluorescence images of MDA-MB-231 acquired by confocal imaging showed decreased mesenchymal shape in 3D after induction of miR-200c for (b) 72 h as well as (c) further increased effects after 168 h compared to included controls.

As a decrease in migration often correlates with changes of the cytoskeleton, we investigated how miR-200c affects the cellular morphology. Hence, immunofluorescence imaging and analysis of the cellular shape was performed. Fig 3a shows a comparison of the actin-structure of MDA-MB-231 with either the Ctrl or the miR-200c construct stimulated with doxycycline for 72 h. While the TRIPZ-Ctrl cells maintained their mesenchymal, spindle-like shape, the miR-200c induction changed the cellular profile towards rounder, uniformly dilated cells as seen in the significant difference of the ratio of widest vs. longest spread of the cell. The three-dimensional shape of the cells was investigated by taking z stacks of confocal images of actin and filamin. Fig 3b and 3c show the 3D images with color coding for height. In line with the previous results, the TRIPZ-Ctrl cells retained their spindle-like structure, after 72h and 168h of doxycycline stimulation. The miR-200c induction caused a gradual transition towards rounder and morphologically flatter cells over time, eventually resulting in evenly flat “pancake” like shape. For better visualization, these effects are presented in a 3D rendering animation of the stacks, shown in the supplemental movies (S2 and S3 Movies). These results together show a strong effect of miR-200c induction on the cellular shape.

### Changed expression of FLNA is observed after a miR-200c knock-out and overexpression

**Fig 4:**
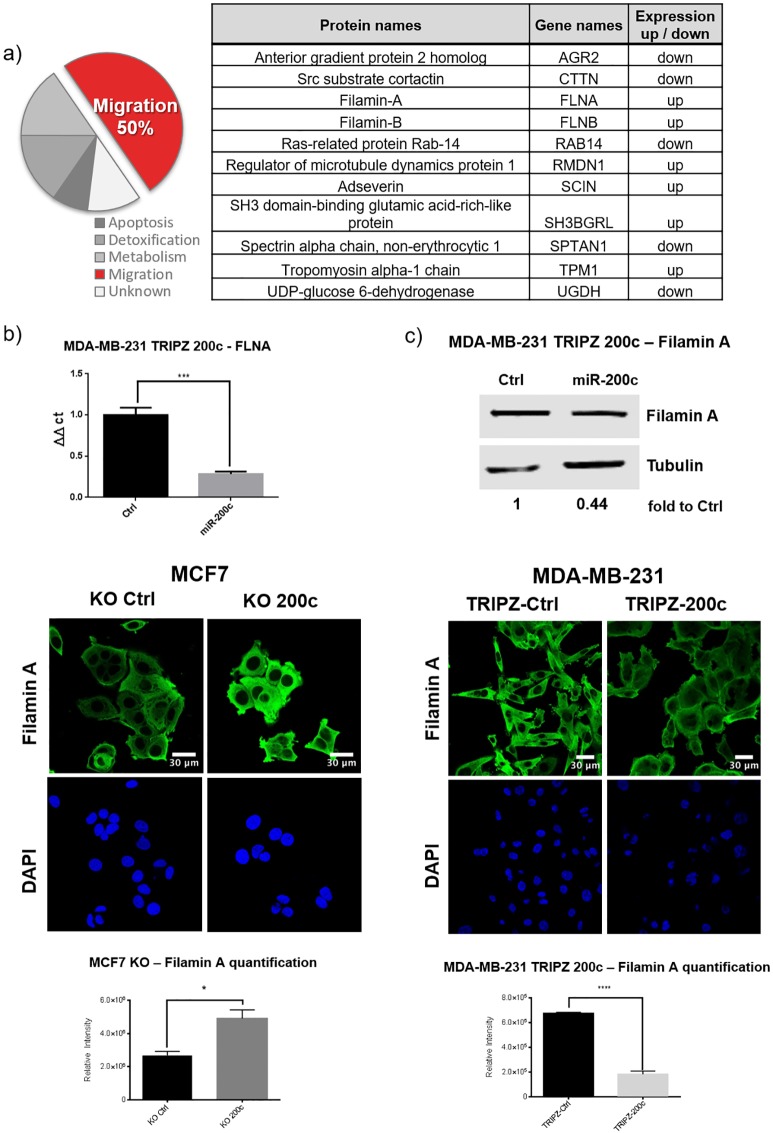
miR-200c regulates migration associated genes such as filamin A. (a) A proteomic analysis of a TALENs knock-out (KO) of miR-200c in MCF7 cells revealed a set of proteins with differential expression, of which 50% are involved in migratory processes and are shown in the table. (RT qPCR showed that after adding 5 μg / ml doxycycline for 48 h the expression of FLNA mRNA (b) as well as filamin A protein(c) (normalized to tubulin) decreased significantly in MDA-MB 231 TRIPZ 200c cells. (d) Immunofluorescence staining of filamin A (green) and DAPI (blue) in MCF7 Ctrl and KO 200c showed significantly increased relative intensity of filamin A, in contrast to (e) the MDA-MB-231 cells which showed a strong decrease in filamin A intensity after induction of miR-200c (all N = 3; error bars indicate standard deviation SD; * p ≤ 0.05, ** p ≤ 0.01, *** p ≤ 0.001, **** p ≥ 0.0001).

A proteomic analysis of a genomic knock out (KO) of miR-200c in MCF7 breast cancer cells was previously reported by our group [26]. There, we showed that more than 50% of all differentially expressed proteins were affiliated to migratory processes (Fig 4a). Out of these proteins, filamin A was one of the prominent and promising targets and therefore chosen for further analysis in this study. To study the biological effect of miR-200c on FLNA the inducible MDA-MB-231 TRIPZ-200c or TRIPZ-Ctrl cells were utilized. In line with studies of the MCF7-200c-KO cells the inverse effects regarding filamin A expression were observed after induction of miR-200c. Here, the mRNA levels of FLNA decreased to 30% and protein expression to 40% compared to doxycycline stimulated TRIPZ-Ctrl cells (Fig 4b and 4c). Additionally, the results were validated in another triple-negative and miR200c-negative cell line by transient transfection of miR-200c. As expected, BT549 cells showed similar results (S1 Fig). Furthermore, an immunofluorescence staining of filamin A was performed in both cell line models. i.e. the MCF7-200c-KO and the MDA-MB-231 TRIPZ-200c. The KO of miR-200c in MCF7 resulted in increased cellular expression of filamin A (Fig 4d), while induction of miR-200c in MDA-MB-231 TRIPZ-200c cells resulted in decreased filamin A protein expression (Fig 4e). Taken together, miR-200c expression showed an indirect proportional relation to filamin A protein as well as mRNA in two complementary breast cancer cell line models.

### miR-200c is regulating FLNA expression via JUN and MRTF-SRF

**Fig 5:**
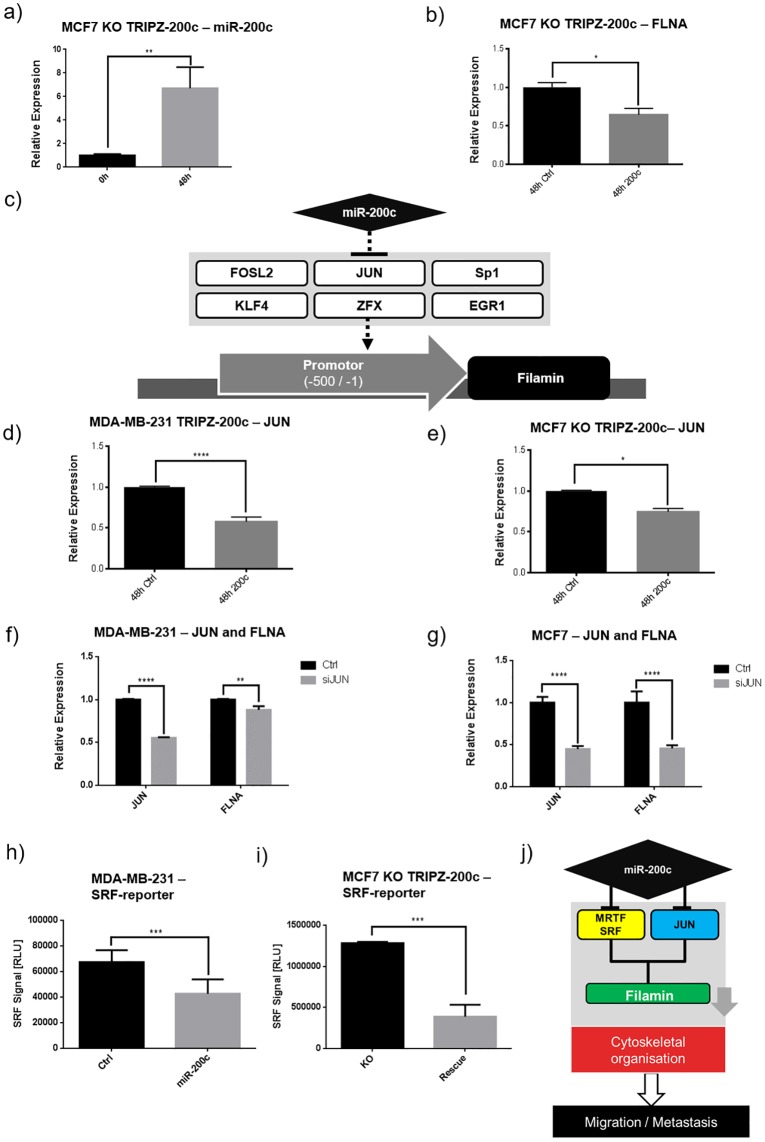
Filamin A is regulated by miR-200c by repression of JUN as well as SRF-MRTF. (a) After introduction of the TRIPZ-200c construct into MCF7 200c KO cells, miR-200c was re-expressed by 48 h of DOX induction and showed (b) significantly decreased FLNA expression. (c) *In silico* analysis of the FLNA promotor revealed 6 transcription factors which have a potential miR-200c binding site. (d) The expression of JUN was significantly decreased 48 h after induction of miR-200c in MDA-MB-231 as well as (e) re-expression in MCF7 compared to the respective TRIPZ-Ctrl cell line. (f, g) Decreased expression of FLNA was verified in MDA-MB-231 and MCF7 after transient transfection with siRNA against JUN (siJUN), compared to scrambled siRNA control (h, i) Induction of miR-200c for 48 h decreased the luciferase signal of a MRT-SRF reporter construct in MDA-MB-231 TRIPZ-200c as well as MCF7 TRIPZ-200c compared to their respective TRIPZ-Ctrl. (j) An alternative mechanism of miR-200c regulation of FLNA is based on reducing the MRT dependent SRF activation as well as the transcription factor jun. (all N = 3; error bars are SD; * p ≤ 0.05, ** p ≤ 0.01, *** p ≤ 0.001, **** p ≥ 0.0001).

To further investigate the mechanism of miR-200c dependent regulation of FLNA, a rescue of miR-200c expression in the MCF7-200c-KO cells was performed, by stably introducing the inducible TRIPZ-200c plasmid. As expected, the induction successfully re-expressed miR-200c and consequently decreased FLNA mRNA (Fig 5a and 5b).

As no miR-200c binding site was predicted *in silico* in the FLNA 3’UTR, other regulatory mechanisms were investigated. First, a promotor analysis was performed, in order to determine transcription factors (TFs) which are potentially regulating FLNA expression and contain an *in silico* predicted miR-200c binding-site (Fig 5c). Four of those TFs were identified by RT-qPCR screening after miR-200c induction in MDA-MB-231 cells (S2 Fig), but a reproducible decrease in expression of these TF was solely detected for JUN (Fig 5d). This result was confirmed in the miR-200c inducible MCF7 KO cells (Fig 5e).

To investigate the effect of JUN on FLNA expression, a siRNA knockdown of JUN was performed in wild type MDA-MB-231 as well as in MCF7 cells and compared to scrambled control siRNA. In both cases, the reduction of JUN mRNA also decreased FLNA mRNA expression (Fig 5f and 5g), with stronger relative effects in MCF7 cells than in MDA-MB-231.

Thus, we suggest JUN as a direct target of miR-200c and thus as putative regulator of FLNA expression.

Another possibility of miR-200c regulating FLNA is via SRF and MRTF. As it is known that that miR-200c regulates SRF and MRTF [23], and also a regulation of FLNA by SRF was predicted in previous studies [35]. Thus, we tested the hypothesis that miR-200c is able to regulate FLNA via the MRTF/SRF axis by transiently introducing pgl4.34, a luciferase reporter for MRTF-dependent SRF activation, into both miR-200c inducible cellular systems. Here, a decrease in luciferase signal upon miR-200c expression was observed in both, MDA-MB-231 and MCF7 cells, compared to their respective doxycycline treated controls (Fig 5h and 5i). These results suggest a regulatory relation between miR-200c and FLNA based on the two different mechanisms, i.e. via transcriptional repression of filamin A through reduced JUN and the regulation by MRTF/SRF (Fig 5j).

## Discussion

miR-200c is a well-established player in different types of cancer, often described as guardian over multiple cancer promoting pathways like metabolic activity and proliferation[16–18], resistance to chemotherapeutics [13–15], and inhibition of migration and EMT [18–22].

In different clinical studies the miR-200c expression correlated with decreasing spread of tumors and better response to therapy of some cancers, as shown in different studies for patients with breast cancer[20, 36, 37]. In the current literature, miR-200c’s effect on metastasis is mainly attributed to the process of EMT, based on preventing ZEB1/2 mediated inhibition of E-cadherin expression and thereby inhibiting the transition of epithelial cells to the mesenchymal phenotype[19, 22, 38]. Another perspective is described by Korpal et al. showing that the expression of the miR-200 family is regulating the expression of Sec23a [25]. By regulating the secretome of cells, which is able to induce MET, the nesting of circulating cancer cells could be initiated, potentially increasing their metastatic capability. In this case, the impact of the expression of miR-200c is dependent on the metastatic status of the primary tumor, decreasing metastatic capabilities by inhibiting EMT during the onset of the process, but increasing potential for manifestation of the metastasis when cell had already spread.

Still, miRNA-200c has shown effects directly on migration of cell lines, which do not express the genetic axis of ZEB-mediated E-cadherin regulation. One issue in the investigation of additional migratory effects of miR-200c lies in distinguishing novel functions from effects based on the prominent ZEB/E-cadherin axis. Therefore, our approach has based on the utilization of two different breast cancer cell lines (MCF7 and MDA-MB-231) which, due to epigenetic predispositions[32–34], ensure absence of ZEB/E-cadherin based effects. Proteomic analysis of a genomic miR-200c KO in MCF7, a high miR-200c expressing epithelial cell line[17] emphasized the importance of miR-200c in migratory processes [26].

In this study, we show the influence of miR-200c on migration based on two “gain of function” cell line models. First, mesenchymal and migratory MDA-MB-231 cells, which lack expression of miR-200c as well as E-cadherin, were transduced with a doxycycline inducible miR-200c expression construct (TRIPZ-200c). This approach ensured to minimize negative effects of transfections on the one hand, and on the other hand allowed for the efficient expression of miR-200c on a long-term scale, rendering the observation of slower processes in cellular remodeling possible. Furthermore, with the same construct, an inducible rescue of the miR-200c expression was performed in the MCF7 miR-200c KO cells.

The induction of miR-200c caused evident changes in the morphology of MDA-MB-231 cells, resulting in extensive remodeling of the cellular architecture as observed already after three days and even further increased after seven days. The resulting flat “pancake” shaped cells appeared to have highly decreased cellular stiffness as well as a lack of distinctive polarization that is commonly seen in spindle-like cells. Similar observations of morphological or “spreading defects” were reported after a FLNA knock-down and were attributed to a deficiency in actin-crosslinking [29, 39].

In contrast to the rather slow full EMT, the effects on speed and run-times of the cells were observed already 48 h hours after miR-200c induction, indicating a direct connection of this axis to the cellular motility. The long-term stimulation showed a change in modality which may be similar to EMT, but a complete transition to an epithelial “cobblestone” phenotype was not observed, possibly due to the lack of development of cellular adhesions by E-cadherin.

Furthermore, the 1D migration assay revealed decreased motility after miR-200c expression by showing changes of all measured parameters the decreased migratory capabilities of miR-200c high expressing cells show to be not based mainly on the absolute velocity, but more on the cells’ inability of polarizing and retaining polarization, as seen by the higher number of cells in temporary rest states or being completely immobile.

Our previously published proteomic analysis of a genomic miR-200c KO in MCF7 disclosed multiple changes in the expression of regulators of migratory processes, of which the effect on filamins A was most prominent [26]. Filamin A is supposed to affect cell motility based on multiple pathways, like the induction of changes in the structure and stiffness of the cell as direct building block in the system[39] or shifts in intracellular signaling[40] and resulting in alteration of different mechanisms important for migration, like the actin-treadmill and formation of focal adhesions[41, 42] ^38^. This important role of filamin A as a capable regulator of cellular migration makes it interesting to investigate how miR-200c regulates filamin A. Especially as we found that induced miR-200c expression resulted in decreased expression of FLNA in both cell systems.

The investigation of the underlying mechanism of FLNA suppression was performed with two *in silico* analyses: The first was not yielding any predicted binding site of miR-200c in the FLNA 3’ UTR, while the other resulted in six potential miR-200c controlled transcription factors. Of the transcription factors, only JUN showed constant repression to miR-200c expression, which is in line with previous studies that identified JUN as potential miR-200c target [43]. JUN expression is necessary for the formation of the AP-1 complex together with c-Fos. Previously, the AP-1 complex was shown to promote tumorigenesis, cancer progression and also regulating cell morphology and migration [44–46]. Our experiments verified JUN promoting FLNA transcription as well as inhibition of JUN by miR-200c’s, resulting in decreased FLNA expression.

A recent study by Jurmeister et al. indicated one further possible regulatory mechanism. They found that miR-200c was inhibiting the MRTF dependent activation of SRF[23]. SRF is a known transcription factor of multiple immediate early genes, including c-fos[47], and therefore an important regulator of cell growth, differentiation and also migration[48, 49] FLNA was previously[35] identified as a target of SRF. Consistently a decreased MRTF-dependent SRF activation was observed after miR-200c induction, indicating an additional axis of miR-200c based regulation of FLNA expression. Furthermore, increased c-Fos expression due to SRF stimulation may also promote the additionally observed JUN-based axis, by providing additional partners for the assembly of the AP-1 complex. Further, FLNA was shown to promote the activity of SRF [50] which may further increase the investigated effects due to this positive feedback loop.

Our data reveal a potential novel route of miR-200c regulating migration, independent of ZEB1/2. The inhibition of cytoskeletal components via miR-200c, like filamin A as shown here, support the role of miR-200c in maintaining the epithelial state and inhibiting the onset of metastasis as possibly important in a wider variety of cancer cells.

## Materials and methods

### Reagents

Puromycin dihydrochloride and doxorubicin hydrochloride were obtained from Sigma (cat. P8833, D1515).

### Cell culture

The MCF7 miR-200c KO cells were grown at 37 °C and 5% CO_2_ in high glucose DMEM (Sigma) supplemented with 10% fetal calf serum (FCS / Gibco). MDA-MB-231 cells were cultured at 37 °C and 0% CO_2_ in L15 (Sigma) containing supplemented with 10% fetal calf serum (FCS / Gibco). All derived cells, i.e. MDA-MB-231 TRIPZ200c and Ctrl, as well as MDA-MB-231 GFP were cultured same as the parental cells. All cells were routinely tested and confirmed as mycoplasm free.

### miRNA-200c knock-out

The miR-200c KO via TALENs was performed as described previously[26].

### Generation of TRIPZ-200c cells and stimulation

As backbone for the TRIPZ-200c construct, the TRIPZ lentiviral inducible shRNA control plasmid (TRIPZ-Ctrl, Thermo Fisher Scientific, #RHS4743) was used. MiR-200c plus 125 bp upstream and downstream flanking genomic sequences, including XhoI and MluI restriction sites was amplified by PCR with the following primers:

Fwd: CTCGAGGCTCACCAGGAAGTGTCCCC
Rev: ACGCGTCCTTGTGCAACGCTCTCAGC.

After the construct was verified by sequencing (GATC Biotech AG), MDA-MB-231 and MCF7 200c KO cells were transducted with the TRIPZ-200c and TRIPZ-Ctrl utilizing a 2^nd^ generation lentiviral system generated with the plasmids pCMV-dR8.2 dvpr and pCMV-VSV-G, which were a gift from Bob Weinberg (Addgene plasmid # 8454 and #8455). After transduction and 48 h selection with 5 μg/ml puromycin, a single cell dilution was performed to generate the monoclonal TRIPZ cell lines MDA-MB-231 TRIPZ-Ctrl, MDA-MB-231 TRIPZ-200c and MCF7 200c KO TRIPZ-200c.

Stimulation of the cells with doxycycline was performed in a concentration of 5 μg/ml in the respective medium for 48 h for mRNA analysis or 72 h for protein analysis. Medium was replaced with fresh, doxycycline containing medium every 48h to compensate for doxycycline degradation.

### miRNA quantitative RT-PCR

qPCR of miRNA was performed as described previously[26], in short: 600,000 cells were harvested and total RNA isolated from cells using miRCURY RNA Isolation Kit (Exiqon). cDNA synthesis was carried out by a miRNA specific reverse transcription and detection with the qScript microRNA cDNA Synthesis Kit and PerfeCta SYBR Green SuperMix (Quanta Biosciences) with RT-PCR detection on a LightCycler 480 (Roche). The expression of miR-200c was normalized to miR-191[51], using the 2^-ΔCT^ or 2^-ΔΔCT^ method.

The following list contains the primers used for analysis of miRNAs:

miR200c: *GCGTAATACTGCCGGGTAAT*; miR-191: GCGCAACGGAATCCCAAAAG;

### Western blot

Cells were cultured in a 6 well plate for 72h after stimulation with doxycycline. Lysis, gel and blotting were performed as described previously[13]. For the detection, the primary antibodies for filamin A (Thermo Fisher, MA5-11705) and tubulin (Sigma, T 9026) were used and diluted by manufacturer’s instructions. For secondary antibody detection, ALEXA FLUOR PLUS 800 (Thermo Fisher, A32730) were used, imaged with the Odyssey Fa and analyzed and quantified by Image Studio Software (LiCor).

### Analysis of transcription factors in promoter regions of found genes

The transcription factor binding sites were published previously[26]

### qPCR validation of mRNA expression

RNA extraction was performed via the Total RNA Kit, peqGOLD (VWR) as by manufacturer’s instructions. The cDNA synthesis was performed using the qScript cDNA synthesis kit (Quanta Bioscience) as by manufacturer’s protocol.

Analysis of expression was performed with the Lightcycler 480 (Roche) and the Universal Probe Library (Roche) with following probe and primer (forward/reverse) combinations, all results were normalized to GAPDH as housekeeper:

FLNA, Probe 32, TCGCTCTCAGGAACAGCA / TTAATTAAAGTCGCAGGCACCTA
JUN, Probe 19, CCAAAGGATAGTGCGATGTTT / CTGTCCCTCTCCACTGCAAC
GAPDH, Probe 45, TCCACTGGCGTCTTCACC / GGCAGAGATGATGACCCTTTT
KLF4, Probe 83, TGACTTTGGGGTTCAGGTG / GTGGAGAAAGATGGGAGCAG
EGR1, Probe 22, AGCCCTACGAGCACCTGAC / GGTTTGGCTGGGGTAACTG
FOSL2, Probe 70, ACGCCGAGTCCTACTCCA / TGAGCCAGGCATATCTACC

### Confocal laser scanning microscopy

Confocal images and 3D stacks were acquired using a Leica TCS SP8 SMD microscope equipped with a 40x HC PL APO oil objective. Pinhole size was adjusted to 1.0 airy units and sequential scanning was performed at 400 Hz. 405nm, 488nm and 561nm laser lines were used for excitation.

### Sample preparation and image analysis

For immunofluorescence staining, cells were fixed for 10 min with 4% EM grade formaldehyde. After 5 min washing with PBS, samples were permeabilized for 10 min with 0.5% TX-100 in PBS. Unspecific binding was blocked by 30 min incubation with 5% BSA (Sigma) at RT. Cells were then incubated overnight (4 °C) with the primary antibody for filamin A diluted according to the manufacturer’s instructions (1:400, Thermo Fisher, MA5-11705). After 3 x 10 min washing with PBS, samples were incubated with secondary antibodies (1:500, AF488 goat-anti-mouse AB_2534069), rhodamine phalloidin (1:300, Sigma-Aldrich) and DAPI (0.5 μg/ml, Sigma-Aldrich) for 1 hour at RT, washed again 3 x 10 min with PBS. All stainings were performed in ibiTreat 8 well μ-slides (ibidi GmbH) coated with fibronectin (Corning). Total fluorescence intensities and nuclear shape factors were quantified using ImageJ v1.52. Z-plane scaled 3D stacks were rendered using the Leica LAS X software platform.

### JUN knockdown

For the knockdown of JUN, a siRNA was used and compared to a negative control (Silencer Select, Thermo Fisher, assay s7659 and control 4390843). Cells were transfected with the K2 transfection reagent (Biontex) according to the manufacturer’s recommendations.

### Luciferase-reporter assay

For analysis of SRF/MRTF signaling, the pgl4.34 Plasmid (Promega, 9PIE135) was used. Transfection was performed in 6-well with cells grown to 80% confluence with K2 transfection reagent (Biontex, Germany) according to the manufacturer’s instructions, into cells stimulated with 5μg/ml doxycycline for 24h. Luciferase measurement was performed 24h after transfection, as described previously[17].

### 1D-Migration

Detailed description of production of the stamps and measurements are published in Schreiber et al. [30] and further described in the supplemental methods (S6 Methods).

The motility parameters are defined as:

***v***_**run**_**:** The run velocity is defined as the mean over the tangential velocity for time points when cells are in the run state *v*_*run*_ = 〈|*v*_*tang*_|〉.
***τ***_**run**_**, *τ***_**rest**_**:** To evaluate the persistence times of run and rest states *τ*_run_ and *τ*_rest_, the survival function *S*(*t*) = P(*T* > *t*) is calculated. *τ*_run_ and *τ*_rest_ are determined by fitting log(*S*(*t*)) by the function $f\left(t\right)=-\frac{1}{\tau }\;t+c$ evaluated at *t* ∈ [2; 16]*h*. Very small times are excluded because defiations from an exponential behavior are observed here. To reduce the effects of the limited time window, only states that start at least 16 h before the end of the corresponding cell track are evaluated, while the fitting range for *S*(*t*) ends at 16 h. The error range given is the 99% confidence interval for the fit.
***P***_**run**_**:** The fraction of time cells spend in the run state is defined as the time cells are in the run state divided by the total time of the trajectories.
***q***: The persistence parameter q is defined as the maximum distance between two points of a cell trajectory divided by the total path length of the trajectory. This is averaged over all cells $q\;=\;\langle \frac{\text{max}\left(\varphi \right)-\text{min}\left(\varphi \right)}{\sum_{i}\left|\varphi_{i}\right|}\rangle$

### Statistical analysis

Results are expressed as the mean ± SD of at least three biological replicas and analyzed using a two-sided student’s t-test, if not stated otherwise. Software GraphPad Prism v6 and SigmaPlot 11 were utilized for the analysis of the data.

## Supporting information

S1 Movie 1D. Migration. A time lapse movie of MBA-MB-231 TRIPZ-Ctrl(left) and TRIPZ-200c (right) cells moving on 1D ring-shaped microlanes.
(AVI)

S2 Movie 3D Animation TRIPZ-Ctrl. A 3D animation of the stacked, rendered images, showing spatial cell shape of TRIPZ-Ctrl.
(AVI)

S3 Movie 3D Animation. A 3D animation of the stacked, rendered images, showing spatial cell shape of TRIPZ-200c.
(AVI)

S1 Fig Effect of miR-200c expression on FLNA in BT549 cells. BT549 cells were transfected with 75 pmol of either miR-200c or scrambled siRNA-Control. After 72 hours cells were harvested for RNA lysis with subsequent qPCR analysis.
(PDF)

S2 Fig Effect of miR-200c expression on mRNA levels of a set of transcription factors. Analysis of different potential transcription factors for FLNA was performed after miR-200c induction. The graphs show the RT-qPCR results at different time points, with no consistent effect for any factor but JUN.
(PDF)

S1 Methods Extended description of 1D Migration patterning. (PDF)

S1 Raw Data (ZIP)
