# Supplementary material for: Inducible microRNA-200c decreases motility of breast cancer cells and reduces filamin A
Source: PLoS One. 2019 Nov 20;14(11):e0224314. doi: 10.1371/journal.pone.0224314 (PMC6867627; doi:10.1371/journal.pone.0224314)
Supplement: S1 Fig — BT549 cells were transfected with 75 pmol of either miR-200c or scrambled siRNA-Control. After 72 hours cells were harvested for RNA lysis with subsequent qPCR analysis. (PDF) [file pone.0224314.s004.pdf]

S1 Fig . Effect of miR-200c expression on FLNA in BT549 cells

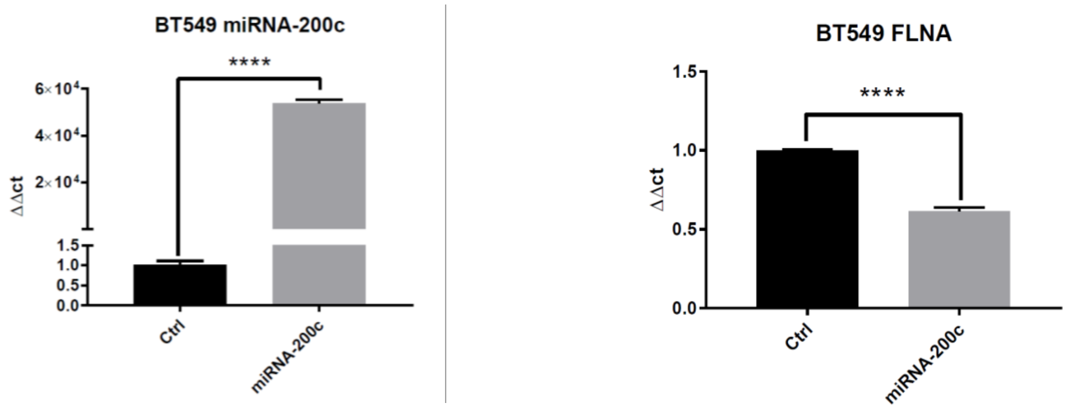

BT549 cells were transfected with 75 pmol of either miR-200c mimic or scrambled siRNA-Control (Dharmacon). After 72 hours cells were harvested for RNA lysis with subsequential qPCR analysis. Two-sided t-test, ****: $p \leq 0.0001$
