# Supplementary material for: Inducible microRNA-200c decreases motility of breast cancer cells and reduces filamin A
Source: PLoS One. 2019 Nov 20;14(11):e0224314. doi: 10.1371/journal.pone.0224314 (PMC6867627; doi:10.1371/journal.pone.0224314)
Supplement: S2 Fig — Analysis of different potential transcription factors for FLNA was performed after miR-200c induction. The graphs show the RT-qPCR results at different time points, with no consistent effect for any factor but JUN. (PDF) [file pone.0224314.s005.pdf]

S2 Fig . Effect of miR-200c expression on mRNA levels of a set of transcription factors A

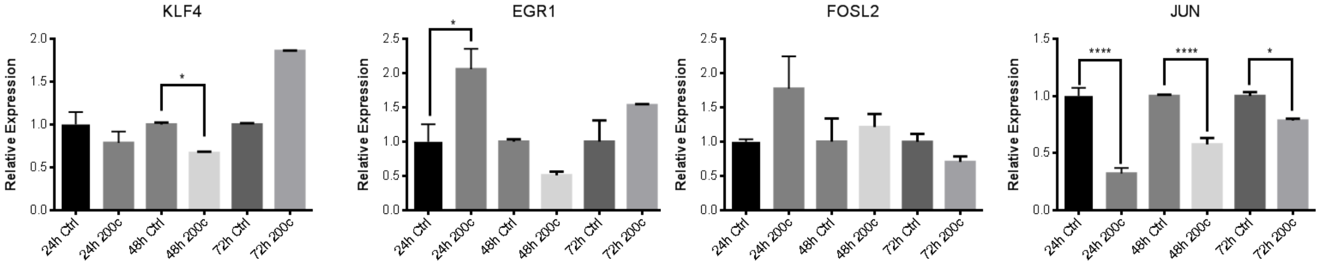

Analysis of different potential transcription factors for FLNA was performed after miR-200c induction. The graphs show the RT-qPCR results at different time points, with no consistent effect for any factor but JUN. *: $p \leq 0.05$; ****: $p \leq 0.0001$
