# Supplementary material for: Inducible microRNA-200c decreases motility of breast cancer cells and reduces filamin A
Source: PLoS One. 2019 Nov 20;14(11):e0224314. doi: 10.1371/journal.pone.0224314 (PMC6867627; doi:10.1371/journal.pone.0224314)
Supplement: S1 Methods — (PDF) [file pone.0224314.s006.pdf]

S1 Fig . Effect of miR-200c expression on FLNA in BT549 cells

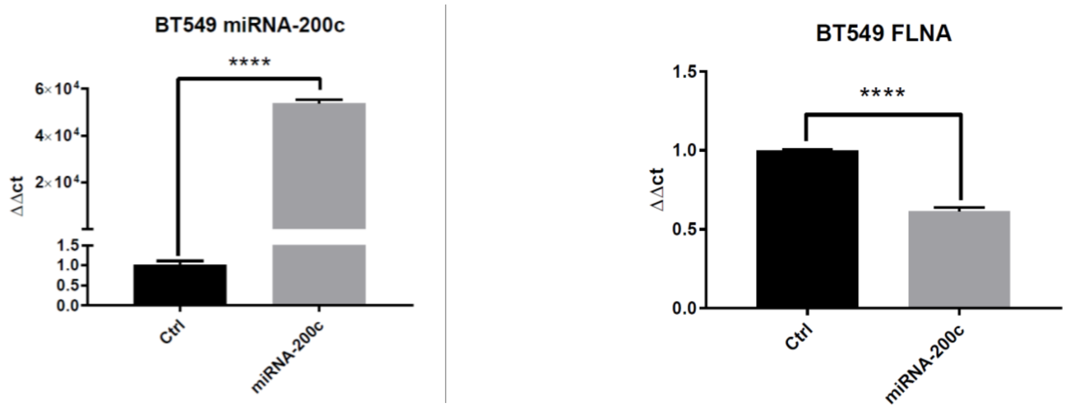

BT549 cells were transfected with 75 pmol of either miR-200c mimic or scrambled siRNA-Control (Dharmacon). After 72 hours cells were harvested for RNA lysis with subsequential qPCR analysis. Two-sided t-test, ****: p ≤ 0.0001

S2 Fig . Effect of miR-200c expression on mRNA levels of a set of transcription factors A

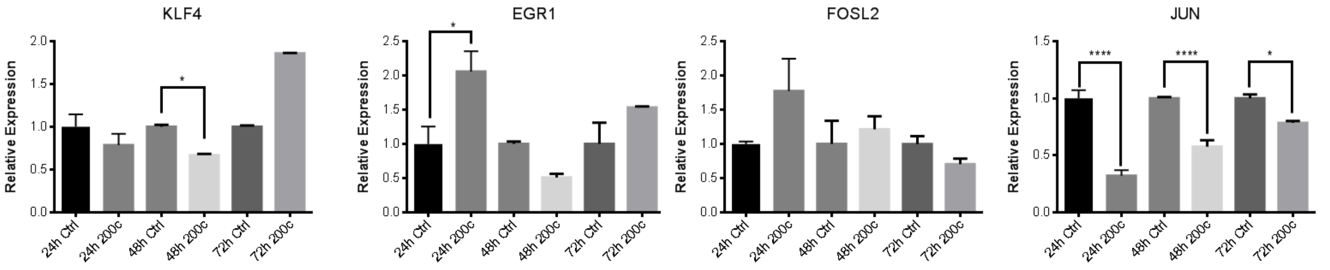

Analysis of different potential transcription factors for FLNA was performed after miR-200c induction. The graphs show the RT-qPCR results at different time points, with no consistent effect for any factor but JUN. *: $p \leq 0.05$; ****: $p \leq 0.0001$

S1 Methods

Micropatterning / 1D Migration
Production of stamps:
To a master for stamp preparation, silicon wafers were coated with TI Prime adhesion promoter and AZ40XT (MicroChemicals) photo-resist. Desired areas were exposed to UV light using laser direct imaging (Protolaser LDI, LPKF). The photoresist was then developed (AZ 826 MIF, MicroChemicals) and silanized (Trichloro(1H,1H,2H,2H-perfluoro-octyl)silane, Sigma-Aldrich). To create the stamp, polydimethylsiloxane (PDMS) monomer and crosslinker (DC 184 elastomer kit, Dow Corning) were mixed in a 10:1 ratio, poured onto the stamp master, degassed in a desiccator, and cured overnight at 50°C.
Microcontact printing:
Microcontact printing was used to produce fibronectin-coated ring-shaped lanes. PDMS stamps were treated with UV light (PSD-UV, novascan) for 5 min. Then the stamps were incubated for 45 min in a solution containing 40 µg/ml fibronectin (Yo proteins) and 10 µg/ml fibronectin labeled with Alexa Fluor 647 (Alexa Fluor NHS Ester, Thermo Fisher Scientific) dissolved in ultrapure water. Next, stamps were washed with ultrapure water, dried and placed on a petri dish (µ-Dish, Ibidi) which had been activated with UV light for 15 min. A droplet of a 2 mg/ml poly-L-lysine-grafted polyethylene glycol (PLL-PEG) (2 kDa PEG chains, SuSoS) solution (dissolved in a solution of 10 mM HEPES and 150 mM NaCl was placed at the edge of the stamps and drawn into the spaces between surface and stamp by capillary action. Stamps were removed and a glass coverslip was placed on the dish surface to ensure complete coverage of the surface with PEG solution. After a 30-min incubation, the coverslip was removed, and the surface was washed three times with phosphate-buffered saline (PBS) and stored in PBS until cells were seeded.
Cell Culture
MDA-MB-231 breast cancer cells were cultured in L15 medium (sigma aldrich) containing 10% TET system approved fetal calf serum (FCS) (Clontech). Cells were incubated at 37°C in a humidified atmosphere. For cell motility measurements, cells were cultured in medium containing 0 or 5 µg/ml Doxocyclin for 44h. Then, about 10,000 cells were seeded per dish (µ-Dish, ibidi). After 3 h, cell medium was replaced with fresh medium containing 25 nM Hoechst 33342 dye (Invitrogen) and measurements were started within 1 hour.
Time-Lapse Microscopy
Scanning time-lapse measurements were performed using an automated inverted microscope (Nikon Ti) equipped with a 10x objective, a LED lamp (Spectra X, lumencor) and a sCMOS camera (pco.edge 4.2, pco). Cells were maintained at 37°C and a humidified atmosphere using a heating chamber (okolab). Phase-contrast and fluorescent images were automatically acquired every 10 min.
Cell Tracking
Cell tracking was performed using the image-processing software ImageJ. Isolated cells confined in the ring-shaped lanes were identified by eye. Fluorescence images of the nuclei were preprocessed by applying a bandpass filter and a threshold for fluorescence intensity, and the centers of mass of the stained nuclei were identified. Cell tracking was stopped in the case of cell division or when cells spanned over the middle part of the ring pattern. Cell tracks shorter than 20 h, as well as tracks of dying or non-moving cells were excluded from further analysis.
Data Analysis
Two-state analysis of tracks:
Track analysis was performed in MATLAB (Mathworks). A circle was fitted to cell position data to find the center of the ring-shaped lane. Switching to polar coordinates, the tangential component of the cell velocity was evaluated as), where indicates the azimuthal cell position at time *i* and *R* indicates the mean radius of the micropattern (*R* = 65 µm). To distinguish run from rest states, a iterative change-point analysis in combination with a classification of cell dynamics in the time intervals between change-points was applied. Change-points were identified when they exceeded a confidence level for the existence of change-points that was calculated via bootstrap analysis of the cumulative sum of the angular velocity. For all intervals between change points this was repeated until no more change-points were found. The resulting intervals were classified into run and rest states by analyzing the mean square displacement. Details are published in Schreiber et al.[cite]
Motility Parameters:
The run velocity is defined as the mean over the tangential velocity for time points when cells are in the run state .
To evaluate the persistence times of run and rest states and , the survival function is calculated. and are determined by fitting by the function evaluated at . Very small times are excluded because defiations from an exponential behavior are observed here. To reduce the effects of the limited time window, only states that start at least 16 h before the end of the corresponding cell track are evaluated, while the fitting range for ends at 16 h. The error range given is the 99% confidence interval for the fit.
: The fraction of time cells spend in the run state is defined as the time cells are in the run state divided by the total time of the trajectories.
q: The persistence parameter q is defined as the maximum distance between two points of a cell trajectory divided by the total path length of the trajectory. This is averaged over all cells
