# Supplementary material for: Inducible microRNA-200c decreases motility of breast cancer cells and reduces filamin A
Source: PLoS One. 2019 Nov 20;14(11):e0224314. doi: 10.1371/journal.pone.0224314 (PMC6867627; doi:10.1371/journal.pone.0224314)
Supplement: S1 Raw Data — (ZIP) [file pone.0224314.s007.zip › Supplement_table_motility.docx]

|  | MDA-MB-231-ctrl no Dox | MDA-MB-231-ctrl 5µg/ml Dox | MDA-MB-231-200c no Dox | MDA-MB-231-200c 5µg/ml Dox |
| --- | --- | --- | --- | --- |
| $v_{Run}$ [µm/h] | 54,8 | 46,9 | 62,8 | 40,4 |
| standard error [µm/h] | [53,5 56,1] | [45,1 48,6] | [61,5 64,1] | [34,1 46,6] |
| $P_{Run}$ | 0,363 | 0,300 | 0,349 | 0,116 |
| standard error | [0,347 0,380] | [0,277 0,322] | [0,337 0,360] | [0,103 0,130] |
| $\tau_{Run}$ [h] | 5,26 | 4,54 | 5,32 | 3,36 |
| 99% conf. Int. [h] | [5,06 5,46] | [4,22 4,87] | [5,05 5,59] | [3,14 3,58] |
| $\tau_{Run}$ [h] | 14,39 | 16,70 | 12,91 | 23,39 |
| 99% conf. Int. [h] | [13,98 14,80] | [15,89 17,51] | [12,33 13,49] | [21,90 24,89] |
| q | 0,367 | 0,338 | 0,377 | 0,212 |
| standard error | [0,356 0.379] | [0,324 0,352] | [0,369 0,384] | [0,201 0,223] |
| N | 291 | 130 | 516 | 117 |

Table S?: Values of parameters quantifying the effect of miR-200c on cell motility of MDA-MB-231 cells shown in Figure 1 g,h. Single cell tracks can be downloaded from: ???
